# Onchocerciasis in the Cameroon–Chad border area after more than 20 years of annual mass ivermectin distribution

**DOI:** 10.1186/s13071-024-06284-8

**Published:** 2024-05-13

**Authors:** Franklin Ayisi, Dziedzom Komi de Souza, Jamie Tallant, Benjamin Didier Biholong, Eric Bertrand Fokam, Daniel Adjei Boakye

**Affiliations:** 1 African Regional Postgraduate Programme in Insect Science (ARPPIS), University of Ghana, PMB LG 59, Legon, Accra, Ghana; 2 National Onchocerciasis Control Programme, Sub Department in Charge of Malaria and Neglected Tropical Diseases, Department of Control of Diseases, Epidemics and Pandemics, Ministry of Public Health, Yaoundé, Cameroon; 3 Department of Parasitology, Noguchi Memorial Institute for Medical Research (NMIMR), College of Health Sciences, University of Ghana, Legon, Accra, Ghana; 4 The END Fund, New York, NY USA; 5 Department of Animal Biology and Conservation, University of Buea, Buea, Cameroon

**Keywords:** Onchocerciasis, Cross-border, *Onchocerca volvulus*, *Simulium damnosum*, MDA, Cameroon

## Abstract

**Background:**

The main vectors of onchocerciasis in Africa are *Simulium damnosum* sensu lato, which transmit the causative agent *Onchocerca volvulus*. The force of transmission is driven by the vector density, hence influencing the disease prevalence and intensity. Onchocerciasis is currently targeted for elimination using mass drug administration (MDA) of ivermectin, a potent microfilaricide. MDA in Cameroon began in 1987 in the Vina Valley, an endemic cross-border area with Chad, known for high vector densities and precontrol endemicity. Evaluations in 2008–2010 in this area showed ongoing transmission, while border areas in Chad were close to interrupting transmission. This study aimed to evaluate transmission in this area after several rounds of MDA since the last evaluation surveys.

**Methods:**

Black flies were collected by human landing catches at seven border sites in Cameroon, twice a week, from August 2021 to March 2022. A fraction of the flies was dissected for parity assessment and identification of *Onchocerca* larval stages. The transmission indices were estimated. Black fly larvae were also collected from the breeding sites at the fly catching sites and identified to species level by cytotaxonomy.

**Results:**

A total of 14,303 female flies were collected, and 6918 were dissected. Of these, 4421 (64.0%) were parous. The total biting rates were high, reaching up to 16,407 bites/person/study period, and transmission potential (third-stage larvae (L3) from head/all L3) were 367/702, 146/506, 51/55, 20/32, 0/3, 0/0, and 0/0 infective larvae/person, respectively, for Mbere-Tchad, Babidan, Hajam/V5, Gor, Djeing, Touboro, and Koinderi. Infectivity rates (L3 from head) were 16.00, 12.75, 5.15, and 4.07 infective females (L3H)/1000 parous flies for Haijam, Mbere-Tchad, Babidan, and Gor, respectively. These values exceed the World Health Organization (WHO) thresholds of ≤ 20 annual transmission potential (ATP) or < 1 infective female/1000 parous females. The major vectors identified were *Simulium damnosum* sensu stricto, *S*. *squamosum*, and for the first time in the area, *S. yahense*.

**Conclusions:**

More than 20 years of MDA has not eliminated onchocerciasis in the study area; hence, this area is a potential source of reintroduction of onchocerciasis in Chad and would require alternative treatment strategies. Many factors such as MDA efficiency, effectiveness of ivermectin, and cytospecies composition may be contributing to transmission persistence.

**Graphical Abstract:**

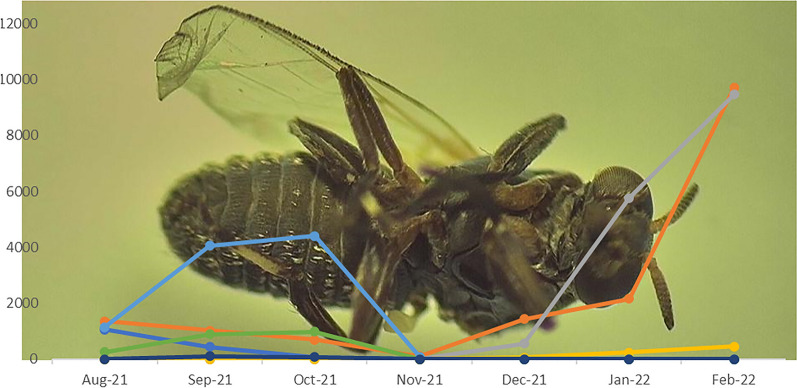

**Supplementary Information:**

The online version contains supplementary material available at 10.1186/s13071-024-06284-8.

## Background

Human onchocerciasis, also known as river blindness, is a debilitating disease with over 220 million people at risk worldwide. More than 99% of infected individuals live in Africa [1]. The causative agent of human onchocerciasis is the filarial worm *Onchocerca volvulus*, which is transmitted by black flies (genus *Simulium*), whose larvae develop in fast-running streams and rivers. In Africa, the main vectors that account for transmission of most cases of onchocerciasis belong to the *Simulium damnosum* complex of sibling species. These are known to disperse an average of 15–20 km from their breeding site, hence the intensity of the disease is highest in villages 10 km or less from a productive breeding site [2].

The burden of human onchocerciasis in Africa has necessitated the formation of large control programs to fight the disease. The Onchocerciasis Control Program (OCP) used large-scale aerial and ground insecticide application against the larval stages of the vectors to control the disease in West Africa. The OCP (1975–2002) successfully controlled onchocerciasis as a public health and socioeconomic problem in most of its member countries [3]. The African Programme for Onchocerciasis Control (APOC) (1995–2015), using mainly mass community-directed treatment with ivermectin (CDTI), achieved more than control of onchocerciasis as a public health problem within its participating countries [4]. The initial objective of the APOC saw a paradigm shift from morbidity control to transmission elimination following the proof of principle of transmission elimination with ivermectin in some foci in West Africa [5, 6]. The Expanded Special Project for the Elimination of Neglected Tropical Diseases in Africa (ESPEN) was set up in 2016 with responsibility to fight against five preventive chemotherapy NTDs, including onchocerciasis. Among the responsibilities of ESPEN are to scale up treatment, build health capacity of countries, improve the supply and effective use of the drugs, and increase resource mobilization to accelerate the control and transmission elimination of these diseases [7].

Many foci in former APOC countries have attained interruption/elimination of transmission (including foci in Ethiopia, Sudan, Uganda, Equatorial Guinea (Bioko Island), and Nigeria) using mass drug administration (MDA) alone or combined with vector control [8]. By 2021, about 2 million people formerly at risk of onchocerciasis in Latin America and Africa had become safe from such risk owing to transmission interruption [1, 9]. This figure will likely increase as several countries have stopped MDA and are currently undertaking posttreatment surveillance (PTS) [9].

The 2021–2030 NTD roadmap targets transmission interruption of human onchocerciasis from 12 countries using ivermectin as the main treatment strategy [10]. Ivermectin drastically reduces microfilaridermia a few days after administration, an effect that lasts for at least 10 months, and also temporarily inhibits the release of new microfilariae by gravid female worms for several months [11]. This leads to a reduction in intensity and prevalence of infection in the community, thereby reducing the number of individuals who could infect the vector [12]. The goal is therefore to administer ivermectin for a period as long as the reproductive lifespan of the adult female worm (12–15 years), preventing transmission until the natural death of the adult female worm. Hence, elimination of onchocerciasis using MDA requires sustained high annual MDA treatment coverage (> 80% of eligible population) for 15–20 years [13, 14]. The North Region of Cameroon (precisely, the Vina Valley–Touboro Health District) was one of the first areas in Africa where mass ivermectin distribution trials were carried out by 1987 [15, 16].

Savanna vectors play a major role in onchocerciasis transmission in the North Region of Cameroon, where *Simulium damnosum* sensu stricto apparently serves as the major vector and *S. sirbanum* likely plays a secondary role [17]. A small number of larvae of forest vectors—*S. squamosum* cytotype A and *S. mengense*—occur periodically in the area [18, 19], possibly contributing to overall transmission. Vector breeding is seasonal in most of the region, where the streams and rivers only flow during the rainy season, except for the perennial rivers Mbere and Vina North, which meet at the border with Chad to form the Logone Occidental River in Chad. Biting rates around the rivers and their tributaries are usually very high [20].

One major challenge with onchocerciasis in West and Central Africa is that the endemic foci are usually large and contiguous, cutting across national and international boundaries [21, 22]. This poses a problem when independent projects or country programs on either side of such boundaries show heterogeneity in their progress toward transmission elimination. This is because an area or country can only be certified for transmission elimination when there is minimal risk of reintroduction of onchocerciasis [13]. The vectors are capable of dispersing up to 20 km around the breeding site, and up to 500 km in migratory movements, making cross-border transmission interruption among the main challenges of onchocerciasis elimination in Africa [23].

The North Region of Cameroon is characterized by savanna bioecology, and consequently the epidemiology of onchocerciasis is mostly manifested by ocular, rather than skin, complications [16]. The region is host to two major hyperendemic foci—the Benue Basin and Vina-Mbere/Touboro [24]. The Vina-Mbere/Touboro focus is a wide hyperendemic onchocerciasis cross-border focus continuous in Chad and the Central African Republic, sustained by the Vina North, Mbere, and Lim (the three that merge to form Logone Occidental) and Pende (called Logone Oriental in Chad) rivers in these countries [25]. Precontrol endemicity in the Vina-Mbere/Touboro focus was extremely high (CMFL of up to 303 microfilariae per skin snip—[26]) compared with levels in all other onchocerciasis endemic areas in the world, with some such extremely highly endemic (holoendemic) villages in Cameroon located close to the border with Chad [4, 26].

Mass ivermectin distribution by health personnel on the Cameroon side of the Vina-Mbere/Touboro focus began in 1987 [16]; however, official records at the Touboro Health District regarding mass drug administration (MDA) with ivermectin are available from 1993 [20]. Communities in the adjacent Rey Bouba and Tchollire Health Districts have had MDA since 1998. Historically, reported, as well as validated, annual treatment coverage in these districts has been very high, exceeding the 80% threshold required for elimination; validated treatment coverage from 2003 to 2009 ranged between 85.4% and 94.1% of the eligible population [20]. Studies have shown that, despite several rounds and high coverage of ivermectin MDA on the Cameroon side of the border, active onchocerciasis transmission is still ongoing [20, 27], while (through MDA) border areas in Chad have either met the stop criteria or are close to doing so [4]. Given that annual MDA has been ongoing since the last evaluations in the area (2008–2010; [20]), we sought to evaluate the current situation of onchocerciasis transmission in this cross-border area between Cameroon and Chad.

## Methods

### Study site

**Fig. 1:**
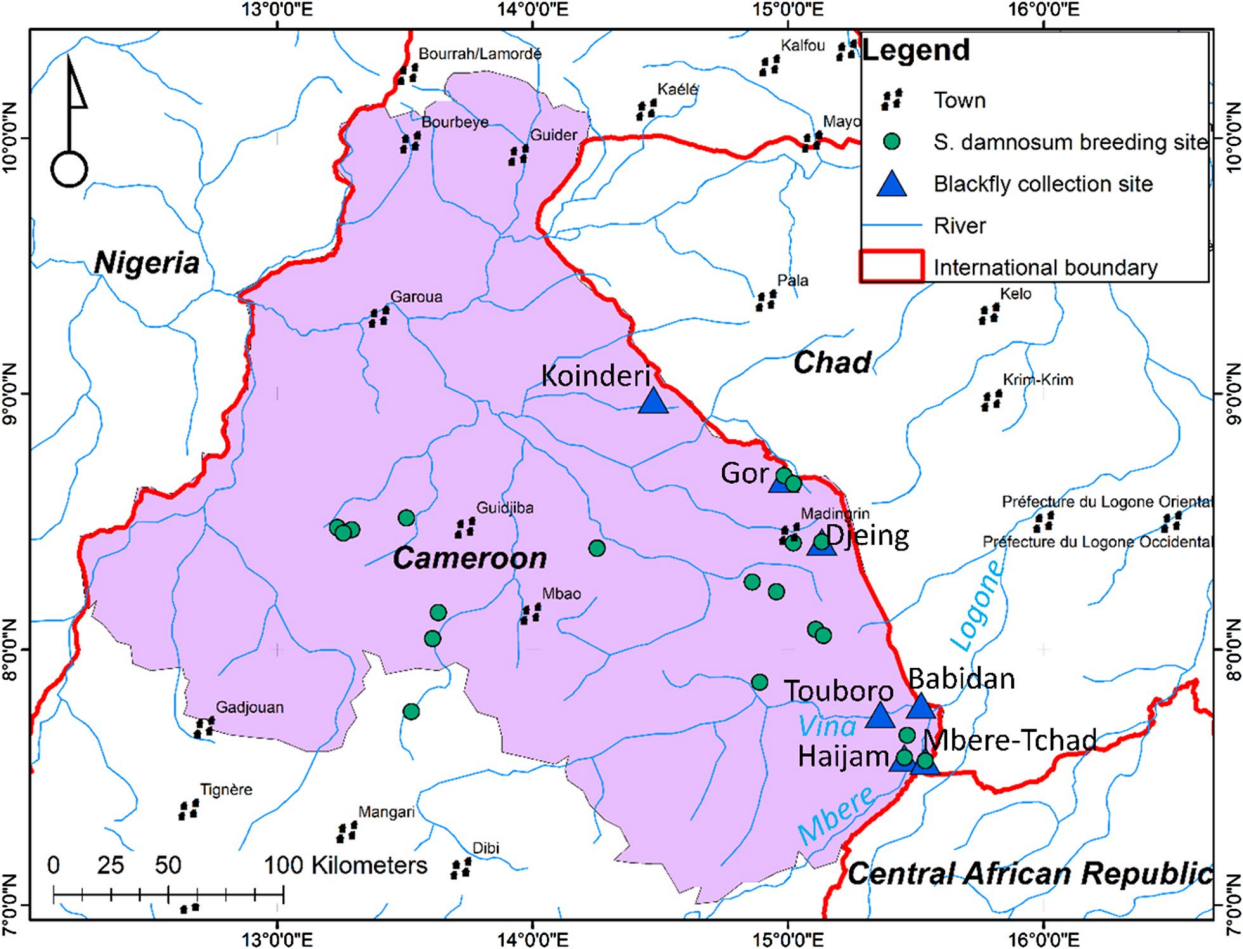
Map of study site showing fly collection points

The study was conducted in seven Cameroon–Chad border sites within three health districts in the North Region of Cameroon. Seven riverside sites near the Koinderi, Djeing, Gor, Babidan, Touboro, Haijam (V5), and Mbere-Tchad communities were selected for black fly collection. Figure 1 shows the study sites, and Table 1 presents the sites in their respective health districts and gives their geographical coordinates and population. The choice of study sites was based on previous transmission data, presence of breeding sites, and consideration of selecting sites across the length of the endemic border between Cameroon and Chad. The area falls within the Sudano-savanna zone with a short unevenly distributed rainy season from June to October and a long dry season from November to May, with slight fluctuations depending on the year.

**Table 1:** Study sites, population, geographical coordinates, and month of recent MDA activity prior to or during the study period

Health district	Village	River	Population^a^	Geographical coordinates	Recent MDA activity dates
Touboro	Haijam/V5	Ebe	3764	7.57717°N, 15.45432°E	June 2021
	Mbere-Tchad	Mbere	1389	7.56532°N, 15.53692°E	April 2020 and December 2021
	Babidan	Vina north	532^b^	7.78244°N, 15.52055°E	May and December 2021
	Touboro (Sodecoton)	Vina north	2124	7.74654°N, 15.36103°E	May and December 2021
Tchollire	Gor (Goindassou 1)	Gelcoma	1086	8.66768°N, 14.98278°E	April 2021
	Djeing	Lidi	1137	8.42090°N, 15.13168°E	April 2021
Rey Bouba	Koinderi	Koinderi	1284	8.97749°N, 14.47348°E	March and November 2021

^a^Population figures are from the 2016 population census done by community drug distributors (CDDs) in preparation for mass drug administration (MDA)

^b^Census figures for 2015, because 2016 census figures were far lower. In Touboro and Gor, which are very large villages, the population mentioned here are those of the quarter/neighborhood closest to the black fly breeding river

North Cameroon is endemic for both animal (*O. ochengi* and *O. ramachandrini*) and human (*O. volvulus*) onchocerciasis, with *S. damnosum* s.l. serving as vector in both cases. The prevalence and intensity of onchocerciasis in the area generally increases from inland toward the border with Chad [26]. Ivermectin treatment began in villages in the area in 1987, with Babidan among the villages that have been treated since 1988 [26]. By 1992, the River Blindness Foundation (RBF) in partnership with the Ministry of Public Health started a mass ivermectin distribution program in the North Region. Available treatment registers documenting MDA in the Touboro Health District were kept from 1993, while those for Tchollire and Rey-Bouba began in 1998 [20]. The African Programme for Onchocerciasis Control (APOC) started funding community-directed treatment with ivermectin (CDTI) projects in the region in 1996.

### Rainfall data

Rainfall data for the four study sites in the Touboro Health District (Touboro, Babidan, Haijam, and Mbere-Tchad) were obtained from the SODECOTON (La Société de développement du coton) regional office at Touboro. Rainfall data for Haijam, Babidan, and Mbere-Tchad were read from nearby stations at Wourlagwe (V5), Bogdibo, and Mbaimboum, respectively. Rainfall data for Touboro were obtained from a weather station at Touboro (radio). We were unable to obtain rainfall data for the other black fly collection sites.

### Collection and dissection of black flies

Recruitment of fly collection volunteers and initial training was done in July 2021 at all the collection sites. Preference was given to volunteers who had participated in previous black fly collection studies in the area. Generally, black flies were collected from August 2021 to March 2022, on two days per week from 7 a.m. to 6 p.m., by two trained volunteers who alternated hourly. Fly collection at a site was discontinued when the river dried off and fly numbers became extremely low. At the Mbere-Tchad site, fly collection was temporarily discontinued after the first 2 weeks of collection in the month of November owing to a drastic drop in fly numbers. Owing to the very low numbers and logistics-related issues, all black flies collected in November were not dissected but stored in alcohol for possible future molecular analyses. For sites that did not have vectors biting at the start of the study (Touboro, Babidan, and Koinderi), fly collection was done for 2 days (7 a.m. to 6 p.m.) every fortnight, until biting was observed to have resumed; the collectors then reverted to the protocol of 2 days per week. The duration of the study took into consideration coverage of both rainy and dry seasons, to span at least the 4–6-month period often used for collection of black flies for entomological evaluation by countries’ onchocerciasis control programs, and also considering logistics. Fly collection volunteers sat on a low stool, exposing their lower limb. Blood-seeking adult female black flies were collected using a locally made mouth aspirator, before they began feeding on the volunteer. Landing rates were therefore considered as biting rates of the black flies. Collected flies were transferred to a makeshift laboratory at the close of each day’s collection for dissection. All or a proportion of the collected flies were dissected, while the rest were preserved in properly labeled bottles containing 90% alcohol for possible future molecular analyses. At least 20% of flies for each hour of collection were dissected and examined for presence of *Onchocerca* larvae according to procedures described by Davies and Crosskey [28]. Briefly, a drop of physiological solution was placed on each individual fly on a glass slide, and the abdomen was opened to observe the ovaries and Malpighian tubules to determine parity. Nulliparous flies were discarded, while parous flies were dissected further in search of larvae of *Onchocerca* species. Infection with any *Onchocerca* larval stage (L1, L2, or L3), and the body section (head, thorax, or abdomen) where the infection was observed, were recorded on the dissection sheet. Third-stage larvae of *O. ramachandrini* were morphologically identified (≥ 900 µm body length), and, together with other nematodes (whether of *Onchocerca* species or not) morphologically different from *O. volvulus*, were excluded from the estimation of transmission indices. When a third-stage larva morphologically indistinguishable from *O. volvulus* was observed in a fly, the larva was harvested and preserved, alongside parts of the fly’s Malpighian tubules as described in Ref. [29], for possible future molecular identification.

### Entomological indices

Parous rates were calculated by multiplying the number of parous flies by 100 and then dividing by the total number of flies dissected. Monthly biting rates (MBRs) were estimated by multiplying the number of black flies collected in the month by the number of days in that month, then dividing by the number of days of capture (collection) in that month. The total biting rates were estimated as the sum of the individual MBRs. Unless otherwise stated, infective flies were considered as those carrying third-stage larva(e) of *Onchocerca* parasites that were morphologically indistinguishable from *O*. *volvulus* in the head (L3H). The monthly transmission potential (MTP) was calculated as the MBR of the month multiplied by the number of L3H for that month, divided by the total number of flies dissected. The transmission potential (TP) for each site was estimated as the sum of the individual MTPs for the entire study period. The infectivity rate was calculated as the total number of infective flies with third-stage larva(e) in the head (L3H) multiplied by 1000 and divided by the total number of parous flies. Also, the parasite load was calculated as the number of infective larvae multiplied by 1000 and divided by the number of parous flies. The transmission potentials (monthly or total) were estimated using third-stage larvae in the head (L3H), and another using all third-stage larvae (L3All). Samples collected in the month of November were not included in the combined entomological indices estimation (for reasons evoked under “Collection and dissection of black flies” section above)—only the MBR were used for plotting the monthly trends.

### Collection and identification of larvae by cytotaxonomy

Black fly larvae were sampled in the North Region during the rainy season (July to October 2021), and sites that were positive for *S. damnosum* s.l. were plotted on the map (Fig. 1). In addition, black fly larvae were surveyed near adult black fly collection sites, and larvae collected were identified by cytotaxonomy as described by Ayisi et al. [17]. Briefly, larval developmental (breeding) sites near the points where adult black flies were collected were prospected, and larvae collected were stored in modified Carnoy’s solution (3 parts absolute ethanol and 1 part glacial acetic acid) and kept cold until identification by cytotaxonomy. Larvae were dissected, and stained with lacto-acetic orcein, and the stained polytene chromosomes were spread on a glass slide by gently squashing between the glass slide and a cover slip. Species identification was based on chromosome inversions and band characteristics as described by previous authors [18, 30, 31].

## Results

### Black fly abundance

**Fig. 2:**
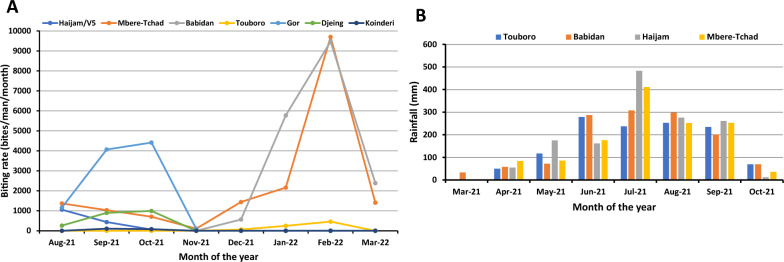
Monthly biting pattern of black flies at the study sites (**A**) and rainfall pattern in 2021 at the four collection sites in the Touboro Health District (**B**)

In all, 14,303 black flies were collected and analyzed (4785 in the rainy season from August to October and 9518 in the dry season from December to March). The total biting rates (Table 2) were high in most of the communities, with higher rates recorded at Mbere-Tchad (16,407 bites/person/study period), Babidan (15,805 bites/person/study period), and Gor (9720 bites/person/study period), compared with biting rates at Haijam/V5 (1,554), Touboro (769), and least at Koinderi (186). No Simuliid flies were seen seeking a blood meal in the rainy season at the Babidan and Touboro sites. Likewise, none was observed at the Haijam/V5, Djeing and Koinderi sites in the dry season (Table 3). Additional file 1: Table S1 shows the MBR, Fig. 2A presents the trends of MBR across the fly collection sites, and Fig. 2B presents the rainfall data of the four collection sites in the Touboro Health District. Biting rates in Gor increased from August to September and slightly increased in October before dropping drastically in November (97.5 bites/person/month). Flies were caught at Mbere-Tchad throughout the study period, with a gradual drop from August to October. This was followed by a dip in November, then by a slow rise up to January and then a sharp increase to reach a peak in February (9702 bites/person/month), followed by a drastic drop in March. Similarly, biting rates in Babidan rose sharply from December, reaching a peak in the month of February and dropping sharply in March. Biting rates at Touboro were low throughout the collection period, rising very slowly from December to reach a peak (458.5 bites/person/month) in February. The fluctuation in biting rate for the other productive rainy season sites was slow throughout the rainy season, with a gradual decrease with decreasing rainfall (Fig. 2A, B). Only 56 black flies were collected from the Koinderi site during the entire study period.

**Table 2:** Transmission indices of onchocerciasis at the study sites

Site	Biting rate (bites/person/period)	Parity (%)	Infective females (L3H)/1000 parous flies	Infective larvae (L3H)/1000 parous flies	TP – L3H (L3All) (infective larvae/man/period)
Haijam/V5	1554	83.9	16	41.21	51 (55)
Mbere-Tchad	16,407	75.7	12.75	29.75	367 (702)
Babidan	15,805	66.3	5.15	11.34	146 (506)
Touboro	769	56.4	0	0	0 (0)
Gor	9720	55.9	4.07	5.08	20 (32)
Djeing	2148	39.4	0	0	0 (3)
Koinderi	186	92.9	0	0	0 (0)

L3All = all third-stage larvae retrieved from both thorax and head of the fly; L3H = third-stage larvae retrieved from the head of the fly. Period = the entire period of the study. Mean values do not include months during which no flies were caught. Transmission potential (TP) was calculated by adding the individual monthly transmission potentials of the months covered in this study (Table 3). Biting rate (BR) was calculated as the sum of the individual monthly biting rates of the months covered in this study

**Table 3:** Monthly transmission potentials (MTPs) and number of infective black flies per site through the study period

	Monthly transmission potential (MTP)/no. of infective flies											
	No. dissected	Rainy season					Dry season					
Site		Parous rate (%)	Aug-21	Sept-21	Oct-21	Total	Parous rate (%)	Dec-21	Jan-22	Feb-22	Mar-22	Total
Haijam/V5	434	83.9	27.1/3	24.0/3	0	51.1/6	0	0/0	0/0	0/0	0/0	0/0
Mbere-Tchad	1866	62.0	3.9/1	42.0/5	27.1/2	73.0/8	86.8	0/0	32.3/2	238.8/7	22.9/1	294.0/10
Babidan	1464	0	0/0	0/0	0/0	0/0	66.3	0/0	73.3/3	72.7/2	0/0	146.0/5
Touboro	110	0	0/0	0/0	0/0	0/0	56.4	0/0	0/0	0/0	0/0	0/0
Gor	2370	41.5	0/0	8.0/2	12.6/2	20.7/4	70.0	0/0	0/0	0/0	0/0	0/0
Djeing	601	39.4	0/0	0/0	0/0	0/0	0	0/0	0/0	0/0	0/0	0/0
Koinderi	56	92.9	0/0	0/0	0/0	0/0	0	0/0	0/0	0/0	0/0	0/0
Total	6901					144.8/18						440.0/15

MTP values were calculated from L3 larvae from the head (L3H) that were morphologically indistinguishable from *Onchocerca volvulus*

### Transmission potentials and parity rates

A total of 6918 flies were dissected, of which 4421 (64.0%) were parous. Table 3 presents the entomological parameters, including parity rates, monthly transmission potentials (MTP), and number of infective flies obtained by considering infective flies as those having third-stage larvae in the head (L3H). The MTP values were high, with the highest values obtained in February at Mbere-Tchad (238 infective bites/person/month), followed by January at Babidan (73.3 infective bites/person/month) and then February at Babidan (72.7 infective bites/person/month). Except for Mbere-Tchad, which recorded MTP values above zero in most of the months during this study (indicating transmission in both seasons), the other sites had zero MTP values either in the dry season (Haijam/V5 and Gor) or in the rainy season (Babidan), indicating seasonal transmission. The MTP at Touboro, Koinderi, and Djeing was zero throughout the study period, indicating that no infective flies (flies with third-stage larva in the head – L3H) were dissected from these sites. The total transmission potentials (L3H/All L3) for the study period (Table 2) were, in decreasing order, 367/702, 146/506, 51/55, 20/32, 0/3, 0/0, and 0/0 infective larvae/person/study period, respectively, for Mbere-Tchad, Babidan, Hajam/V5, Gor, Djeing, Touboro, and Koinderi. At Mbere-Tchad, which had transmission throughout the study period, transmission was higher in the dry season (294 infective bites/person/season) than rainy season (73 infective bites/person/season). Also, the overall seasonal parous rates were high at all the study sites, with the highest recorded in the rainy season at Koinderi (92.9%) followed by the dry season at Mbere-Tchad (86.8%) and then the rainy season at Haijam/V5 (83.9%). The parity rates of female black flies at Mbere-Tchad were higher in the dry season (86.8%) than in the rainy season (62.0%).

### Effect of recent MDA on transmission

**Fig. 3:**
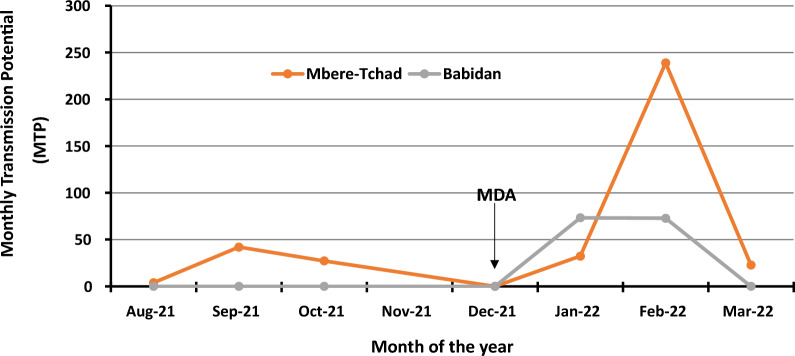
Effect of recent MDA on transmission at Mbere-Tchad and Babidan

Table 1 presents the study sites and the months during which recent mass ivermectin distribution (MDA) was started in the villages. Mbere-Tchad had delays between MDAs, as it took almost 1 year 8 months between the MDA in 2020 and that in 2021. Meanwhile, Babidan, Touboro, and Koinderi carried out biannual MDA in 2021. Ivermectin MDA was administered in April 2020 and December 2021 for Mbere-Tchad and in May 2021 and December 2021 for Babidan. Monthly transmission potentials (MTPs) for the Mbere-Tchad and Babidan sites increased in January 2022 despite having MDA in December, and it either further increased (Mbere-Tchad) or remained constant (Babidan) in February, before dropping rapidly in March (Fig. 3; Table 3).

### Infectivity rates and parasite load

Thirty-three flies were infective, carrying 73 third-stage filarial larvae in the head (L3H) that were morphologically indistinguishable from *O. volvulus* (Table 3). An additional 57 third-stage larvae were found in the thorax (L3-thorax) of dissected flies. The infectivity rates ranged from 0 at Koinderi, Djeing, and Touboro to 16 infective females (L3H)/1000 parous flies at Haijam. Mbere-Tchad, Babidan, and Gor had infectivity rates of 12.75, 5.15, and 4.07 infective females (L3H)/1000 parous flies, respectively (Table 2). The parasite loads were 41.21, 29.75, 11.34, and 5.08 L3H/1000 parous flies at Haijam, Mbere-Tchad, Babidan, and Gor, respectively (Table 2). Two flies at Djeing were infected, one containing a second-stage larva in the thorax (1L2) and the other infected with one third-stage and one second-stage larvae in the thorax (1L3-thorax and 1L2).

### ***Simulium damnosum*** sensu lato breeding sites and species identified by cytotaxonomy

The Gor, Djeing, and Haijam breeding sites were positive for *S. damnosum* s.l. only during the rainy season, as the streams at these sites dried up in the dry season. On the other hand, the Babidan and Touboro breeding sites were positive for *S. damnosum* s.l. only during the dry season, with dense *S. damnosum* s.l. larval populations throughout the dry season period (January to March). The Mbere-Tchad site was positive for *S. damnosum* s.l. in both seasons. No *S. damnosum* s.l. larva were observed at the Koinderi black fly collection site. Three cytoforms of the *S. damnosum* complex (*S. damnosum* s.s., *S*. *squamosum*, and *S. yahense*) were identified by cytotaxonomy from the breeding sites near the black fly collection points (Table 4). One larva from Djeing (Mayo Lidi) could not be classified as either *S. damnosum* s.s. or *S. sirbanum* owing to the presence of a complex inversion (IIL-C/C.8.3), hence we designated this as *S. damnosum*/*sirbanum*. The dominant cytoform at the study sites was *S. damnosum* s.s. (savanna cytoform), except for the Mbere-Tchad site, which was dominated by forest cytoforms—*S. yahense* and *S*. *squamosum*. This is the first report of *S. yahense* from northern Cameroon.

**Table 4:** Cytospecies of *Simulium damnosum* sensu lato identified at the study sites

Site/river	Species (number of larvae)
Haijam(V5)/Ebe	*S. damn* s.s (06)
Mbere-Tchad/Mbere	*S. yahense* (12), *S. squam* (02), *S. damn* s.s (01)
Babidan/Vina south	*S. damn* s.s (11), *S. sirb* (02), *S. damn/sirb* (02)
Touboro/ Vina south	*S. damn* s.s (10)
Gor/Gelcoma	*S. damn* s.s (17)
Djeing/Lidi	*S. damn* s.s (10), *S. damn/sirb* (01)

*S. damn* s.s = *Simulium damnosum* sensu stricto; *S. squam* = *S. squamosum*; *S. damn/sirb* = *Simulium damnosum/sirbanum*. No *S. damnosum* larvae were found at the Koinderi site

## Discussion

The objective of this study was to evaluate the status of transmission of human onchocerciasis in Cameroon’s cross-border area with Chad, and to assess any possible risk this area may pose to the elimination program in Chad. Areas in the North administrative region of Cameroon, including the Vina Valley at the Cameroon–Chad border, are known for their historical extremely high prevalence of human onchocerciasis and very high black fly biting rates [4, 20, 27, 32–34], maintained by breeding sites in the Faro, Vina North, Mayo Rey, Mbere, and Benoue rivers and their tributaries. The present study has shown that black fly vectors are still abundant in this area, with the observed high vector densities contributing to the high force of transmission of onchocerciasis in this area. There was definite seasonality in the human biting activity of black flies in the study area. Similar to previous observations [17], black flies breeding and biting in the dry season are exclusively concentrated around the major perennial rivers (Vina North and Mbere), while in the rainy season, they mostly occur in the seasonal tributaries of these rivers. Hence, we recommend taking this observation into consideration when planning vector surveillance or control in the region, as well as when scheduling CDTI (as treatment is recommended to be done just before the peak transmission period, which is generally assumed to coincide with peak fly densities).

Our study found that only the Mbere River had black fly productivity throughout the study period, which peaked in the dry season (February). It was remarkable that sites in the perennial Vina North River (Babidan and Touboro) had zero black fly catches during the rainy season and early dry season months of the present study (August to November). In fact, the first black flies at the Vina North River sites (Touboro and Babidan) were collected in late December 2021 after over 4 months of zero black fly catches. Meanwhile, previous studies in sites around the Vina North River recorded year-round biting by black flies [27, 33, 34].

In addition, black fly biting densities at Touboro (Vina Bridge) have drastically dropped [769 bites/person/period (August to March)] compared with baseline data (26,100 bites/person/year) collected at this site between 1976 and 1979 [33, 35]. Although the figures are not directly comparable owing to the fact that the duration of the present study was less than a year (8 months), the difference is still quite significant, given that the present study covered the months known (from historical data from 1984 to 2012) to have the greatest black fly densities in the Vina North River [27]. One reason for the large differences in biting rates between previous studies [33–35] and the present study is the fact that the present study neither observed black flies breeding (juvenile stages) nor adult black flies biting throughout the rainy season and the early dry season months. Meanwhile, previous authors [33–35] observed perennial breeding and black fly activity at the Touboro site, with high onchocerciasis transmission in July [35].

Furthermore, unlike the Babidan site, which observed rapid growth of black fly densities following resumption of breeding in December 2021, in the Vina North River, biting rates at the Touboro site remained, surprisingly, very low throughout the study period. This was despite the dense population of larvae of *S. damnosum* s.s. observed at the breeding sites (from January to March) near the fly catching point around the Vina Bridge. The near absence of biting adult black flies at this site (despite the presence of dense larval breeding) may be due to growth in the human population in Touboro, leading to encroachment of open-air town conditions to the Vina North River, causing a reduction in black fly biting rates [35]. Another reason could be that the *S. damnosum* s.s. at this site have a strong preference for cattle rather than human hosts, as previously observed in this area [36]. Therefore, the large herds of cattle that were constantly available by the river banks during the dry season, owing to seasonal transhumance, may have attracted the black flies emerging from the breeding site away from the human fly collection volunteers.

The parity rates of black flies were also quite high, implying high survival rates of the flies and hence higher tendency to transmit onchocerciasis in their lifetime. At Mbere-Tchad, where black flies were collected across both seasons, we observed higher parity rates (86.8%) in the high black fly productive dry season than the low black fly productive rainy season (62.0%). This is contrary to the expectation that large black fly productivity should result in lower parity rates, due to large numbers of newly emerged flies in the population [37]. It is possible that some of the biting black flies caught around this area may be invading flies coming from seasonal streams and rivers that had dried up, as shown in other studies [38].

It is generally considered, in onchocerciasis control, that infective black flies are those having third-stage larvae in the head. The force of transmission (the annual transmission potential, ATP) estimated from such criteria is said to be unable to sustain onchocerciasis transmission when the value is below 20 [13]. Estimates of transmission potentials using L3H in this study exceeded this WHO threshold value at the Mbere-Tchad (367), Babidan (146), and Haijam (51) sites within the Touboro Health District. The transmission potential at the Gor site (20), in the Tchollire Health District, was borderline to the threshold. These results indicate that there is ongoing transmission in the Cameroon–Chad border areas in Cameroon.

It has been suggested that the ATP estimated from all third-stage larvae (whether in the head or other parts of the insect—L3All) is likely a better indicator of transmission than using only L3 in the head [39]. The previous evaluation done from 2008–2010 in the study area [20] reported annual transmission potentials (estimated from all L3 larvae—from thorax and head—and without distinguishing cattle or human origin of L3s) of 543.4 infective bites/person/year for sites in the Touboro Health District, and 20.7 and 40.0 infective bites/person/year for sites within the Tchollire Health District. More than a decade later, the present study recorded still very high transmission potentials calculated from all L3s from sites in the Touboro Health District such as Mbere-Tchad (702) and Babidan (506). This is despite a shorter fly collection period in the present study (6–8 months) compared with the 2008–2010 study (1 year). One explanation for this apparently stable rate of transmission (after more than a decade of additional annual MDA) may be due to differences in choice of fly collection sites, given that only one of the sites in the previous study (Babidan) was selected in this present study—highlighting the importance of evaluation site selection. Nevertheless, there may be other factors contributing to the observed results than just differences in evaluation sites.

The World Health Organization has set a cutoff value for transmission interruption of less than one infective black fly in 1000 parous flies (< 1/1000), or less than two infective flies in 2000 flies (assuming a 50% parous rate), per transmission zone [13]. The higher infectivity and parous rates recorded in this study indicate that transmission is still ongoing in the area, especially in the Touboro Health District. One possible explanation for the delay in progress toward transmission interruption despite decades of ivermectin MDA in the area has been suggested to be due to the initial very high precontrol transmission intensity [4, 20]. This study cannot refute this possibility, although other factors, such as suboptimal response to ivermectin by the adult worm [38] and inefficiencies in the CDTI process (excluding treatment coverage that historically is higher than the required threshold of 80%—[20]), may equally be contributing to the persistence in transmission. This is supported by the high transmission indices (transmission potential and infectivity rates) recorded in the first two months following ivermectin distribution. These high values suggest three possibilities: (i) the actual transmission rates were very high such that the ivermectin distribution could only successfully reduce the rates to the observed levels; (ii) there were individuals in these communities who still had high skin microfilariae after treatment—either because they missed the recent MDA or they actually participated but were carriers of *O. volvulus* that responds poorly to ivermectin; (iii) the infective larvae (although morphologically similar to *O. volvulus*) were coming from animal (cattle) hosts and hence not affected by treatment of human population. It is equally likely that a combination of these possibilities may be at play in these communities. It is therefore important to carry out further studies to elucidate the factors contributing to the observed high transmission. So far, studies in Cameroon have reported deficiencies in CDTI [40, 41], lower clearance of microfilaridermia, and higher repopulation rates [42] as factors contributing to persistent onchocerciasis transmission after several years of MDA.

The parasite loads at Haijam/V5 (41.21) and Mbere-Tchad (29.75) were relatively high compared with the other sites (11.34 and 5.08 L3H/1000 parous flies for Babidan and Gor). However, the values were closer to those observed by Cheke and Garms [39] for savanna species (*S*. *damnosum*/*S*. *sirbanum*), compared with that observed for *S. yahense* in evergreen forest areas of Liberia. Nonetheless, relatively closer parasite loads (67 and 89) were observed for *S. yahense* at moist semideciduous forest sites in Liberia [39]. The parasite load may therefore not give a good explanation for the cytotaxonomic observation of *S. yahense* as major vector breeding at the Mbere-Tchad site. However, it may be that the vectorial capacity of this forest vector is affected by the disease pattern as related to forest and savanna ecological zones, or that both species (forest and savanna) are presently contributing to transmission at these two close-by sites (Haijam/V5 and Mbere-Tchad), a reason why their parasite loads are relatively higher. In any case, the observation of *S. yahense*, a cytospecies that has never been reported in this area, calls for more vigilance in the onchocerciasis control/elimination drive for Cameroon and Chad.

## Conclusions

This study has shown that more than 20 years of mass ivermectin distribution around the Chad border areas in Cameroon has not interrupted onchocerciasis transmission. Based on WHO recommendations, the Chad program cannot be certified to have achieved transmission elimination unless this border transmission situation in Cameroon is resolved. The initial very high force of transmission may be one reason for the persistence in transmission, but it is possible that suboptimal response of *O. volvulus* to ivermectin, inefficient CDTI implementation, and vectorial factors may also be contributing to the present situation. We therefore recommend that these possible confounding factors be analyzed further to identify any role they may be playing, in order to mitigate the situation. This study represents the first ever report of *S. yahense* from northern Cameroon, occurring around the Chad border. This very efficient forest vector cytoform, which has been associated with blinding onchocerciasis, may be playing a significant role in the transmission observed in the area. Meanwhile, we propose that coupling ground larviciding to current interventions may be helpful given the high biting rates and seasonality of transmission in the area.

### Supplementary Information

**Additional file 1: Table S1**. Entomological indicators for all seven study sites.

## Data Availability

The datasets analyzed during the current study are available from the corresponding author on reasonable request.

## Abbreviations

ATP: Annual transmission potential
WHO: World Health Organization
CDTI: Community-directed treatment with ivermectin
MDA: Mass drug administration
OCP: Onchocerciasis Control Program
APOC: African Programme for Onchocerciasis Control
ESPEN: Expanded Special Project for the Elimination of Neglected Tropical Diseases
NTDs: Neglected tropical diseases
PTS: Posttreatment surveillance
CMFL: Community microfilarial load
MTP: Monthly transmission potential
L3H: Infective larva in the head
MBR: Monthly biting rate
