# Supplementary material for: Onchocerciasis in the Cameroon–Chad border area after more than 20 years of annual mass ivermectin distribution
Source: Parasit Vectors. 2024 May 13;17:219. doi: 10.1186/s13071-024-06284-8 (PMC11089731; doi:10.1186/s13071-024-06284-8)
Supplement: Supplementary file 1 — Additional file 1: Table S1. Entomological indicators for all seven study sites. [file 13071_2024_6284_MOESM1_ESM.docx]

Entomological indicators in the seven study sites during the rainy season

|  |  | Haidjam | Mbere-T. | Babidan | Touboro | Gor | Djeing | Koinderi |
| --- | --- | --- | --- | --- | --- | --- | --- | --- |
| Aug. 2021 | No. Catching days | 8 | 8 | 4 | 4 | 8 | 8 | 4 |
|  | No. Collected / No. dissected flies | 273 / 273 | 353/353 | 0 / NA | 0 / NA | 299/299 | 67/67 | 0/NA |
|  | MBR | 1057.9 | 1367.9 | 0 | 0 | 1158.6 | 259.6 | 0 |
|  | Parous rate (%) | 86.4 | 45.3 | NA | NA | 33.8 | 28.4 | NA |
|  | No. Infective flies (L3H) | 3 | 1 | NA | NA | 0 | 0 | NA |
|  | Infective females (L3H)/1000 parous flies | 13 | 6 | NA | NA | 0 | 0 | NA |
|  | No. All L3s / No. L3Hs | 8/7 | 3/1 | NA | NA | 0/0 | NA | NA |
|  | MTP (L3H) | 27.1 | 3.9 | 0 | 0 | 0 | 0 | 0 |
| Sept. 2021 | No. Catching days | 10 | 10 | 4 | 4 | 10 | 10 | 10 |
|  | No. Collected / No. dissected flies | 145/145 | 344/344 | 0 / NA | 0 / NA | 1356/1021 | 299/278 | 35/35 |
|  | MBR | 435.0 | 1032.0 | 0 | 0 | 4068.0 | 897.0 | 105 |
|  | Parous rate (%) | 80.0 | 79.9 | NA | NA | 38.5 | 46.4 | 88.6 |
|  | No. Infective flies (L3H) | 3 | 5 | NA | NA | 2 | 0 | 0 |
|  | Infective females (L3H)/1000 parous flies | 26 | 18 | NA | NA | 5 | 0 | 0 |
|  | No. All L3s / No. L3Hs | 8/8 | 19/14 | NA | NA | 2/2 | 1/0 | NA |
|  | MTP (L3H) | 24.0 | 42.0 | 0 | 0 | 8.0 | 0 | 0 |
| Oct. 2021 | No. Catching days | 8 | 8 | 4 | 4 | 8 | 8 | 8 |
|  | No. Collected / No. dissected flies | 16/16 | 182/182 | 0 / NA | 0 / NA | 1139/1050 | 256/256 | 21/21 |
|  | MBR | 62.0 | 705.3 | 0 | 0 | 4413.6 | 992.0 | 81.4 |
|  | Parous rate (%) | 75.0 | 60.4 | NA | NA | 46.7 | 34.8 | 100.0 |
|  | No. Infective flies (L3H) | 0 | 2 | NA | NA | 2 | 0 | 0 |
|  | Infective females (L3H)/1000 parous flies | 0 | 18 | NA | NA | 4 | 0 | 0 |
|  | No. All L3s / No. L3Hs | 0 | 7/7 | NA | NA | 4/3 | NA | NA |
|  | MTP (L3H) | 0 | 27.1 | 0 | 0 | 12.6 | 0 | 0 |
| Aug.-Oct. 2021 | No. Catching days | 26 | 26 | 12 | 12 | 26 | 26 | 26 |
|  | No. Collected / No. dissected flies | 434/434 | 879/879 | 0 / NA | 0 / NA | 2794/2370 | 622/601 | 56/56 |
|  | Mean MBR | 518.3 | 1035.1 | 0 | 0 | 3213.4 | 695.2 | 62.1 |
|  | Parous rate (%) | 83.9 | 62.0 | NA | NA | 41.5 | 39.4 | 92.9 |
|  | No. Infective flies (L3H) | 6 | 8 | NA | NA | 4 | 0 | 0 |
|  | Infective females (L3H)/1000 parous flies | 16 | 15 | NA | NA | 4 | 0 | 0 |
|  | No. All L3s / No. L3Hs | 16/15 | 29/22 | NA | NA | 6/5 | 1/0 | NA |
|  | Mean MTP (L3H) | 17.0 | 24.3 | 0 | 0 | 10.9 | 0 | 0 |

Entomological indicators in the seven study sites during the dry season

|  |  | Haidjam | Mbere-T. | Babidan | Touboro | Gor | Djeing | Koinderi |
| --- | --- | --- | --- | --- | --- | --- | --- | --- |
| Nov. 2021 | No. Catching days | 0 | 4 | 4 | 4 | 8 | 4 | 0 |
|  | No. Collected / No. dissected flies | NA | 14/0 | 0 / NA | 0 / NA | 26/0 | 0/NA | NA |
|  | MBR | NA | 105 | 0 | 0 | 97.5 | 0 | NA |
|  | Parous rate (%) | NA | NA | NA | NA | NA | NA | NA |
|  | No. Infective flies (L3H) | NA | NA | NA | NA | NA | NA | NA |
|  | Infective females (L3H)/1000 parous flies | NA | NA | NA | NA | NA | NA | NA |
|  | No. All L3s / No. L3Hs | NA | NA | NA | NA | NA | NA | NA |
|  | MTP (L3H) | NA | NA | 0 | 0 | NA | 0 | NA |
| Dec. 2021 | No. Catching days | 0 | 10 | 10 | 10 | 0 | 0 | 0 |
|  | No. Collected / No. dissected flies | NA | 464/93 | 182 / 37 | 20 / 20 | NA | NA | NA |
|  | MBR | NA | 1438.4 | 564.2 | 62 | NA | NA | NA |
|  | Parous rate (%) | NA | 71.3 | 69.7 | 50 | NA | NA | NA |
|  | No. Infective flies (L3H) | NA | 0 | 0 | 0 | NA | NA | NA |
|  | Infective females (L3H)/1000 parous flies | NA | NA | NA | NA | NA | NA | NA |
|  | No. All L3s / No. L3Hs | NA | NA | NA | NA | NA | NA | NA |
|  | MTP (L3H) | NA | 0 | 0 | 0 | NA | NA | NA |
| Jan. 2022 | No. Catching days | 0 | 8 | 8 | 8 | 0 | 0 | 0 |
|  | No. Collected / No. dissected flies | NA | 558 / 134 | 1490 / 394 | 64 / 40 | NA | NA | NA |
|  | MBR | NA | 2162.3 | 5773.8 | 248 | NA | NA | NA |
|  | Parous rate (%) | NA | 89.6 | 75.6 | 45 | NA | NA | NA |
|  | No. Infective flies (L3H) | NA | 2 | 3 | 0 | NA | NA | NA |
|  | Infective females (L3H)/1000 parous flies | NA | 17 | 10 | NA | NA | NA | NA |
|  | No. All L3s / No. L3Hs | NA | 10/2 | 23/5 | NA | NA | NA | NA |
|  | MTP (L3H) | NA | 32.3 | 73.3 | 0 | NA | NA | NA |
| Feb. 2022 | No. Catching days | 0 | 8 | 8 | 8 | 0 | 0 | 0 |
|  | No. Collected / No. dissected flies | NA | 2772/650 | 2705 / 781 | 131 / 50 | NA | NA | NA |
|  | MBR | NA | 9702 | 9467.5 | 458.5 | NA | NA | NA |
|  | Parous rate (%) | NA | 88.2 | 69.1 | 68 | NA | NA | NA |
|  | No. Infective flies (L3H) | NA | 7 | 2 | 0 | NA | NA | NA |
|  | Infective females (L3H)/1000 parous flies | NA | 12 | 4 | NA | NA | NA | NA |
|  | No. All L3s / No. L3Hs | NA | 26/16 | 14/6 | NA | NA | NA | NA |
|  | MTP (L3H) | NA | 238.8 | 72.7 | 0 | NA | NA | NA |
| Mar. 2022 | No. Catching days | 0 | 8 | 10 | 0 | 0 | 0 | 0 |
|  | No. Collected / No. dissected flies | NA | 363/123 | 769 / 256 | NA | NA | NA | NA |
|  | MBR | NA | 1406.6 | 2383.9 | NA | NA | NA | NA |
|  | Parous rate (%) | NA | 95.1 | 42.6 | NA | NA | NA | NA |
|  | No. Infective flies (L3H) | NA | 1 | 0 | NA | NA | NA | NA |
|  | Infective females (L3H)/1000 parous flies | NA | 9 | NA | NA | NA | NA | NA |
|  | No. All L3s / No. L3Hs | NA | 5/2 | NA | NA | NA | NA | NA |
|  | MTP (L3H) | NA | 22.9 | 0 | NA | NA | NA | NA |
| Dec.-Mar. 2022 | No. Catching days | 0 | 34 | 36 | 26 | 0 | 0 | 0 |
|  | No. Collected / No. dissected flies | NA | 4157/1000 | 5146/1468 | 215/110 | NA | NA | NA |
|  | Mean MBR | NA | 3677.3 | 4547.4 | 256.2 | NA | NA | NA |
|  | Parous rate (%) | NA | 86.8 | 66.3 | 56.4 | NA | NA | NA |
|  | No. Infective flies (L3H) | NA | 10 | 5 | 0 | NA | NA | NA |
|  | Infective females (L3H)/1000 parous flies | NA | 12 | 5 | NA | NA | NA | NA |
|  | No. All L3s / No. HL3s | NA | 41/20 | 37/11 | NA | NA | NA | NA |
|  | Mean MTP (L3H) | NA | 73.5 | 36.5 | 0 | NA | NA | NA |
